# Dietary Probiotics or Synbiotics Supplementation During Gestation, Lactation, and Nursery Periods Modifies Colonic Microbiota, Antioxidant Capacity, and Immune Function in Weaned Piglets

**DOI:** 10.3389/fvets.2020.597832

**Published:** 2020-12-14

**Authors:** Kai Wang, Qian Zhu, Xiangfeng Kong, Mingtong Song, Md. Abul Kalam Azad, Liang Xiong, Yuzhong Zheng, Qinghua He

**Affiliations:** ^1^Department of Food Science and Engineering, College of Chemistry and Environmental Engineering, Shenzhen University, Shenzhen, China; ^2^CAS Key Laboratory of Agro-ecological Processes in Subtropical Regions, Hunan Provincial Key Laboratory of Animal Nutritional Physiology and Metabolic Process, National Engineering Laboratory for Pollution Control and Waste Utilization in Livestock and Poultry Production, Institute of Subtropical Agriculture, Chinese Academy of Sciences, Changsha, China; ^3^Key Laboratory of Optoelectronic Devices and Systems of Ministry of Education and Guangdong Province, College of Optoelectronic Engineering, Shenzhen University, Shenzhen, China; ^4^School of Food Engineering and Biotechnology, Hanshan Normal University, Chaozhou, China

**Keywords:** antioxidant capacity, immune function, intestinal microbiota, probiotics, synbiotics, weaned piglets

## Abstract

This study was conducted to investigate the effect of dietary probiotics or synbiotics supplementation on colonic microbiota, antioxidant capacity, and immune function in weaned piglets. A total of 64 pregnant Bama mini-sows and then 128 of their weaned piglets were randomly assigned into control group, antibiotics group, probiotics group, or synbiotics group. The results showed that colonic *Firmicutes* and *Bifidobacterium* abundances in the probiotics group and total bacteria, *Bacteroidetes*, and *Lactobacillus* abundances in the synbiotics group were increased (*P* < 0.05), while *Escherichia coli* abundance in the synbiotics group was decreased (*P* = 0.061) compared with the control group. *Firmicutes, Bifidobacterium*, and total bacteria abundances were increased (*P* < 0.05) in the probiotics and synbiotics groups compared with the antibiotics group. Probiotics supplementation up-regulated (*P* < 0.05) the mRNA expression of GPR109A compared with the control and antibiotics groups. Dietary probiotics or synbiotics supplementation improved the antioxidant capacity by increasing (*P* < 0.05) the colonic CAT, GSH-Px, SOD, and T-AOC levels and plasma CAT, GSH, GSH-Px, and SOD levels and by decreasing (*P* < 0.05) the colonic and plasma MDA and H_2_O_2_ levels. Compared to the control group, the colonic IL-10, IFN-α, and sIgA concentrations and plasma IgA and IgM concentrations were significantly increased (*P* < 0.05) in the probiotics and synbiotics groups. Spearman's correlation analysis showed that the changed colonic microbiota, such as *Lactobacillus* and *Bifidobacterium* were correlated with the alteration of antioxidant indexes, cytokines, and immunoglobulins. In conclusion, dietary probiotics or synbiotics supplementation during gestation, lactation, and nursery periods could be used as an alternative for antibiotics in terms of gut health of weaned piglets.

## Introduction

Weaning is a stressful event in the life cycle of pigs and is associated with the rapid shift in gut microbiota composition, reducing antioxidant ability and intestinal functions, eventually resulting in indigestion, diarrhea incidence, growth retardation, and even death (1, 2). Antibiotics have been widely used as a feed additive to prevent piglets from an intestinal infection and enhance growth performance. However, overuse of antibiotics has the risk of antibiotic-resistant microbes and environmental pollution, which have attracted more attention globally (3). Therefore, effective alternatives for antibiotics from natural sources are urgently needed to surmount its adverse effect.

Maternal microbiota can be transmitted to offspring through direct contact during parturition or breast milk during lactation, which can influence the neonates' gut microbiota colonization (4, 5). The early colonization of intestinal microbiota is an essential stimulus for the development and shaping of immunity in the mucosa (6). Probiotics refer to live microorganisms, which are administered in adequate amounts and confer microbial balance, particularly in the gastrointestinal tract (7). Several studies demonstrated that the provision of probiotics could optimize the intestinal microbiota composition, present significant antioxidant abilities, and improve immune function in weaned piglets (8–10). When xylo-oligosaccharide (XOS) are used together with probiotics, they are called synbiotics, which can reconstruct intestinal microbiota by promoting the proliferation of probiotics bacteria (11). In humans, maternal probiotics consumption during the gestation and lactation periods has been demonstrated as a strategy to influence infant health (6). Maternal dietary supplementation with probiotic species, including *Lactobacillus helveticus* BGRA43, *Lactobacillus fermentum* BGHI14, and *Streptococcus thermophilus* BGVLJ1-44 can improve microbiota diversity in neonatal piglets (12). In addition, dietary supplementation with *Bacillus subtilis* C-3102 to sows during gestation and lactation periods and to progeny after weaning modified the fecal microbiota population in sows and nursery piglets (13). Our previous studies also demonstrated that dietary supplementation with compound probiotics (*L. Plantarum B90* and *S. cerevisiae P11*) or synbiotics (compound probiotics + XOS) during pregnancy and lactation periods can improve piglet's survival and lipid metabolism by altering gut microbiota diversity and composition (14, 15). However, the long-term effect of providing probiotics or synbiotics to sows and their offspring on the colonic microbiota, antioxidant capacity, and immune function in weaned piglets is still unclear.

In this context, we hypothesized that dietary probiotics or synbiotics supplementation might enhance the antioxidant capacity and immune function by modifying the intestinal microbiota community in weaned piglets. Therefore, we aimed to investigate the effect of dietary probiotics or synbiotics supplementation during gestation, lactation, and nursery periods on the colonic microbiota community, antioxidant capacity, and immune function in weaned Bama mini-pigs. The Bama mini-pigs is an indigenous “fatty” strain in China with smaller sizes and delicious meat. Thus, this study could also provide a potential approach to improve the intestinal health of Chinese indigenous pig breeds.

## Materials and Methods

### Animals, Experimental Design, and Diets

The animal experiment was conducted in a mini-pig farm of Goat Chong located in Shimen Town, Changde City, Hunan Province, China. A total of 64 pregnant Bama mini-pigs in parity 3–5 with a similar body condition were randomly allocated to one of four groups (16 sows per group), representing the control group (basal diet), antibiotics group (basal diet + 50 g/t virginiamycin), probiotics group (basal diet + 200 mL/d probiotics fermentation broth per pig), and synbiotics group (basal diet + 200 mL/d compound probiotics fermentation broth per pig + 500 g/t XOS), respectively. The probiotics fermentation broth was purchased from Hunan Lifeng Biotechnology Co., Ltd., and contained viable *L. Plantarum* B90 BNCC1.12934 ≥ 1.0 × 10^8^ CFU/g and *S. cerevisiae* P11 BNCC2.3854 ≥ 0.2 × 10^8^ CFU/g. The XOS contained xylobiose, xylotriose, and xylotetraose (accounting for ≥35%), and was provided by Shandong Longlive Biotechnology Co., Ltd. From mating to day 110 of gestation, the sows were assigned to individual cages (2.2 × 0.6 m). Thereafter, the sows were moved to individual farrowing pens (2.2 × 1.8 m) with a heated floor pad for piglets and *ad libitum* access to water for both sows and piglets until weaning.

After weaning at 28 days of age, two piglets close to the average body weight were selected per litter, and four piglets in the same group were fed in one pen, and eight pens (replicates) per treatment (*n* = 8). After transferred to a nursing facility, the weaned piglets were treated with the same additive as their sows for 1 month: control group (basal diet), antibiotics group (basal diet + 40 g/t virginiamycin), probiotics group (basal diet + 30 mL/d probiotics fermentation broth per pig), and synbiotics group (basal diet + 30 mL/d probiotics fermentation broth per pig + 250 g/t XOS), respectively. The experimental design schematic was shown in Supplementary Figure 1.

The trail period was from sow mating to offspring 30 days after weaning. The sows were fed with pregnant diets from day 1 after mating to day 104 of gestation, and fed with lactating diets from day 105 of gestation to weaning. The basal diet composition and nutrient levels for the sows and piglets met the Chinese nutrient requirements of swine in China (NY/T65-2004) (16), and the premixes met the NRC recommended requirements (NRC, 2012) (Tables 1, 2). Animals were provided with water *ad libitum* throughout the trial. The pigs were fed twice daily (At 8:00 and 17:00) and changed with their body condition.

**Table 1:** Composition and nutrient levels of basal diets for sows (air-dry basis; %).

**Items**	**Pregnant diet**	**Lactating diet**
**Ingredients, %**		
Corn	37.50	66.00
Soybean meal	9.50	25.00
Wheat bran	14.00	5.00
Barley	25.00	
Soybean hull	10.00	
Pregnant sows' premix^a^	4.00	
Lactating sows' premix^b^		4.00
Total	100.00	100.00
Nutrient levels^c^		
Digestible energy, MJ/Kg	12.55	13.87
Crude protein	12.82	16.30
Crude fiber	4.56	2.87
SID Lys	0.48	0.75
SID Met + Cys	0.43	0.51
SID Thr	0.37	0.53
SID Trp	0.13	0.17
Calcium	0.62	0.65
Phosphorus	0.47	0.50

a *Pregnant sows' premix provided the following per kg of diets: CaHPO_4_·2H_2_O 10 g, NaCl 4 g, CuSO_4_·5H_2_O 80 mg, FeSO_4_·H_2_O 360 mg, ZnSO_4_·H_2_O 240 mg, MnSO_4_·H_2_O 100 mg, MgSO_4_·7H_2_O 1 g, 1% ICl 50 mg, 1% Na_2_SeO_3_ 36 mg, 1% CoCl_2_ 16 mg, NaHCO_3_ 1.4 g, VA 10 000 IU, VD_3_ 1 800 IU, VE 20 mg, VK_3_ 2.4 mg, VB_1_ 1.6 mg, VB_2_ 6 mg, VB_6_ 1.6 mg, VB_12_ 0.024 mg, folic acid 1.2 mg, nicotinamide 20 mg, pantothenic acid 12 mg, biotin 0.12 mg, ferrous glycinate 100 mg, choline chloride 1 g, phytase 200 mg, fruity 80 mg, and limestone 12 g*.

b *Lactating sows' premix provided the following per kg of the diet: CaHPO_4_·2H_2_O 10 g, NaCl 4 g, CuSO_4_·5H_2_O 80 mg, FeSO_4_·H_2_O 360 mg, ZnSO_4_·H_2_O 240 mg, MnSO_4_·H_2_O 100 mg, 1% ICl 50 mg, 1% Na_2_SeO_3_ 36 mg, 1% CoCl_2_ 16 mg, NaHCO_3_ 1.4 g, VA 10 000 IU, VD_3_ 1 800 IU, VE 20 mg, VK_3_ 2.4 mg, VB_1_ 1.6 mg, VB_2_ 6 mg, VB_6_ 1.6 mg, VB_12_ 0.024 mg, folic acid 1.2 mg, nicotinamide 20 mg, pantothenic acid 12 mg, biotin 0.12 mg, Lysine 1.5 g, ferrous glycinate 100 mg, choline chloride 1 g, phytase 200 mg, fruity 80 mg, and limestone 12 g*.

c *Calculated nutrient levels using values for feed ingredients from the NRC (2012) (17)*.

**Table 2:** Composition and nutrient levels of the basal diet for weaned piglets.

**Ingredients**	**Ratio, %**	**Nutrients**	**Levels, %^b^**
Corn	54.92	DE, MJ/kg	13.50
Soybean meal	22.00	CP	16.13
Wheat bran	10.13	Ca	0.44
Rice bran	8.95	TP	0.50
Premix^a^	4.00	Lys	1.40
Total	100.00	Met + Cys	0.69
		Thr	0.78

a *Premix provided the following per kilogram of diets: enzymic preparation 1.2 g, VA 26 000 IU,VD_3_ 10 000 IU,VE 70 IU,VK_3_ 10 mg,VB_1_ 10 mg,VB_2_ 25 mg,VB_6_ 10 mg,VB_12_ 0.075 mg, biotin 0.4 mg, folic acid 5 mg, nicotinamide 100 mg, pantothenic 50 mg, choline 1 600 mg, flavoring agent 500 mg, edulcorant 300 mg, acidulating agent 5 g, Cu (as CuSO_4_·5H_2_O) 230 mg, Mn (as MnSO_4_·H_2_O) 97 mg, Zn (as ZnSO_4_·H_2_O) 218 mg, Fe (as FeSO_4_·H_2_O) 165 mg, I (as Ca(IO_3_)_2_) 0.3 mg, Se (as Na_2_SeO_3_) 0.3 mg, Co (as CoSO_4_·xH_2_O) 0.4 mg, glucose 2.1 g, antioxidants 0.4 g, antimildew agent 1 g, Ca (as CaHPO_4_ and CaCO_3_) 3.42 g, P (as CaHPO_4_) 1.155 g*.

b *Calculated nutrient levels using values for feed ingredients from the NRC (2012) (17)*.

### Samples Collection

On day 30 after weaning, eight piglets from each group (one piglet per pen) were selected to collect blood samples (10 mL per piglet) from the precaval vein into heparinized vacuum tubes. The plasma was separated by centrifugation at 3,500 g for 10 min at 4°C and immediately stored at −20°C for analysis of cytokines, immunoglobulin (Ig), antioxidant enzymes, H_2_O_2_, and MDA. The sampled animals were exsanguinated after electrical stunning (120 V, 200 Hz). The colonic contents (middle position) were collected and stored at −80°C for microbiota composition analysis. Colon tissue (middle position) was excised and their contents were flushed with ice-cold phosphate buffer solution. Then the mucosa scrapings (~2 g) were collected and immediately frozen in liquid nitrogen and stored at −80°C for analysis of cytokines, sIgA, antioxidant enzymes, H_2_O_2_, and MDA.

### Microbiota DNA Extraction and Real-Time Quantitative PCR Analysis

Total genomic DNA was extracted from 300 mg fresh colonic luminal contents by using Mag-Bind® Stool DNA Kit (Omega, Guangzhou, China) after chemical and mechanical disruptions. The abundances of total bacteria, *Bacteroidetes, Bifidobacterium, Clostridium cluster* IV, *Escherichia coli*, and *Lactobacillus*, were quantified by real-time polymerase chain reaction (RT-PCR) using specific primers (Supplementary Table 1) and SYBR Green Premix (Takara Biotechnology, Dalian, China) in the LightCycler® 480 II Real-Time PCR System (Roche, Basel, Swiss). The standard curves of each bacterial gene were generated with 10-fold serial dilutions of the 16S rRNA genes amplified from the respective target strains. The PCR reaction mixture and PCR amplification condition were following the instructions of SYBR Green Premix (Takara Biotechnology, Dalian, China). The melting curves were checked after amplification to ensure single product amplification of consistent melting temperature. Quantification of 16S rRNA gene copies in each sample was performed in triplicate, and the mean gene expression 2^−ΔΔCt^ value was calculated. The data were expressed as Lg gene copies/g contents (18).

### Colonic Mucosa RNA Extraction and Real-Time Quantitative PCR Analysis

Total RNA of the colonic mucosa was extracted using the TRIZOL reagent (Magen, Guangzhou, China) according to the manufacturers' protocol. The total RNA (1,000 ng) was reversely transcribed into cDNA using a PrimeScrip RT reagent kit with gDNA Eraser (TaKaRa Biotechnology, Dalian, China). The two-step qRT-PCR analysis was performed on the LightCycler® 480 II Real-Time PCR System (Roche, Basel, Swiss) with the SYBR® Premix Ex Taq™ (TaKaRa Biotechnology, Dalian, China). Pig-specific primers were designed and synthesized by Sangon Biotech (Shanghai) Co., Ltd., China (Supplementary Table 2). The RT-qPCR was performed in a 10 μL reaction system, including 0.25 μL of each primer, 5.0 μL SYBR® Premix Ex Taq, 2.0 μL cDNA, and 2.5 μL of double-distilled water. The PCR cycling conditions referred to the instructions of SYBR Green Premix. The relative changes in gene expression were calculated by the 2^−ΔΔt^ method normalized to housekeeping gene β-actin.

### Colonic Mucosa and Plasma Redox Status Analysis

Colonic mucosa samples (about 0.1000 g) were homogenized with ice-cold physiologic saline (1:9, w/v) and centrifuged at 2,000 g for 20 min at 4°C, and the supernatant was obtained for further analyses. The colonic protein concentration was quantified using the Pierce BCA Protein Assay Kit (CoWin Biosciences, Jiangsu, China).

The plasma and colonic mucosa antioxidant enzymes (CAT, SOD, GSH, and GSH-Px) and MDA levels were measured using the commercially available ELISA assay kit (Jiangsu Yutong Biological Technology, Jiangsu, China), as per the manufacturer's instructions. The T-AOC activity and H_2_O_2_ concentration were detected following the manufacturer's instructions (Nanjing Jiancheng Bioengineering Institute, Nanjing, China). The absorbance values were read on a Multiscan Spectrum Spectrophotometer (Tecan, Infinite M200 Pro, Switzerland). All the redox-related indexes of colonic mucosa were normalized to the total protein concentration of each sample.

### Colonic Mucosa and Plasma Immunoglobulins and Cytokines Analysis

Colonic mucosa samples were prepared as mentioned above. The total protein content of colonic mucous was quantified using the Pierce BCA Protein Assay Kit (CoWin Biosciences, Jiangsu, China). The immunoglobulins and cytokines in the plasma and colonic tissues were measured using the commercially available ELISA kits (Jiangsu Yutong Biological Technology, Jiangsu, China), as per the manufacturer's instructions. The absorbance levels were read on a Multiscan Spectrum Spectrophotometer (Tecan, Infinite M200 Pro, Switzerland). The concentrations of Ig and cytokines in the intestinal mucosa were standardized to the total protein in each sample.

### Statistical Analyses

Data were analyzed by one-way ANOVA and multiple comparisons with the Bonferroni-correction method using SPSS 22.0 statistical package (SPSS Inc., Chicago, IL). All data were expressed as the means ± standard error of the mean (SEM). The significance value and a trend toward differences were set at levels of *P* < 0.05 and 0.05 ≤ *P* < 0.10, respectively. The R package of “Hmisc” was used for calculating the Spearman's correlation coefficient.

## Results

### Microbiota Abundances in Colonic Contents

**Figure 1:**
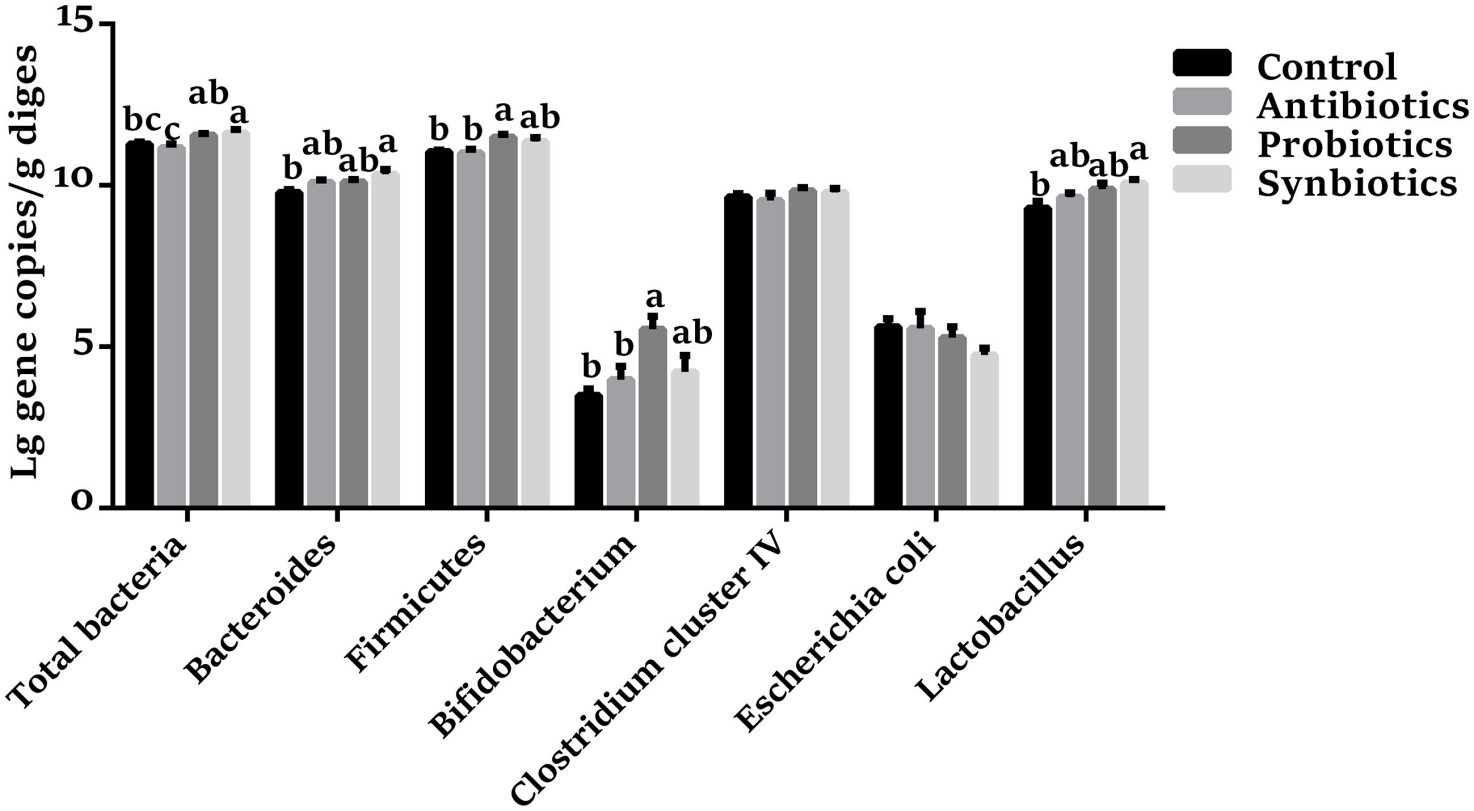
Effect of dietary probiotics or synbiotics supplementation during gestation, lactation, and nursery periods on the copy numbers [Lg (copies/g)] of major bacteria community in the colon contents of weaned piglets. Data are means ± SEM (*n* = 8). a–c represents statistically significant differences among the four groups (*P* < 0.05).

As shown in Figure 1, compared with the control group, the abundances of *Firmicutes* and *Bifidobacterium* were higher (*P* < 0.05), and the abundances of total bacteria (*P* = 0.073) and *Bacteroidetes* (*P* = 0.067) were trends toward an increase in the probiotics group; dietary synbiotics supplementation increased (*P* < 0.05) the abundances of total bacteria, *Bacteroidetes*, and *Lactobacillus*, as well as *Firmicutes* (*P* = 0.083), while decreased (*P* = 0.061) the abundance of *E. coli*. In addition, compared with the antibiotics group, the probiotics supplementation increased (*P* < 0.05) the abundances of total bacteria, *Firmicutes*, and *Bifidobacterium*; the synbiotics supplementation increased (*P* < 0.05) the abundances of total bacteria and tended to increase (*P* = 0.079) the abundance of *Lactobacillus*. However, antibiotics treatment did not affect the determined bacterial species in piglets.

### SCFA Transporters and Receptors in Colonic Mucosa

As shown in Table 3, dietary probiotics supplementation upregulated (*P* < 0.05) the mRNA expression of GPR109A compared with the control and antibiotics groups. However, there were no significant differences in the mRNA expression of FFAR2, SLC5A8, SLC16A1, and SLC27A4 among the four groups.

**Table 3:** Effect of dietary probiotics or synbiotics supplementation during gestation, lactation, and nursery periods on the mRNA expression of short-chain fatty acid (SCFA) transporters and receptors in the colonic mucosa of weaned piglets.

	**Dietary treatment**					***P*-values**
	**Control**	**Antibiotics**	**Probiotics**	**Synbiotics**	**SEM**	
FFAR2	1.13	1.33	1.50	1.14	0.12	0.685
SLC5A8	1.28	1.27	1.13	2.06	0.23	0.543
SLC16A1	1.16	1.18	0.93	1.56	0.11	0.310
SLC27A4	1.03	1.32	1.07	1.13	0.05	0.180
GPR109A	1.08^b^	1.42^b^	2.81^a^	4.65^ab^	0.35	0.001

*Data are expressed as means ± SEM (n = 8). a,b represents statistically significant differences among the four groups (P < 0.05)*.

### Redox Status in Colonic Mucosa

**Figure 2:**
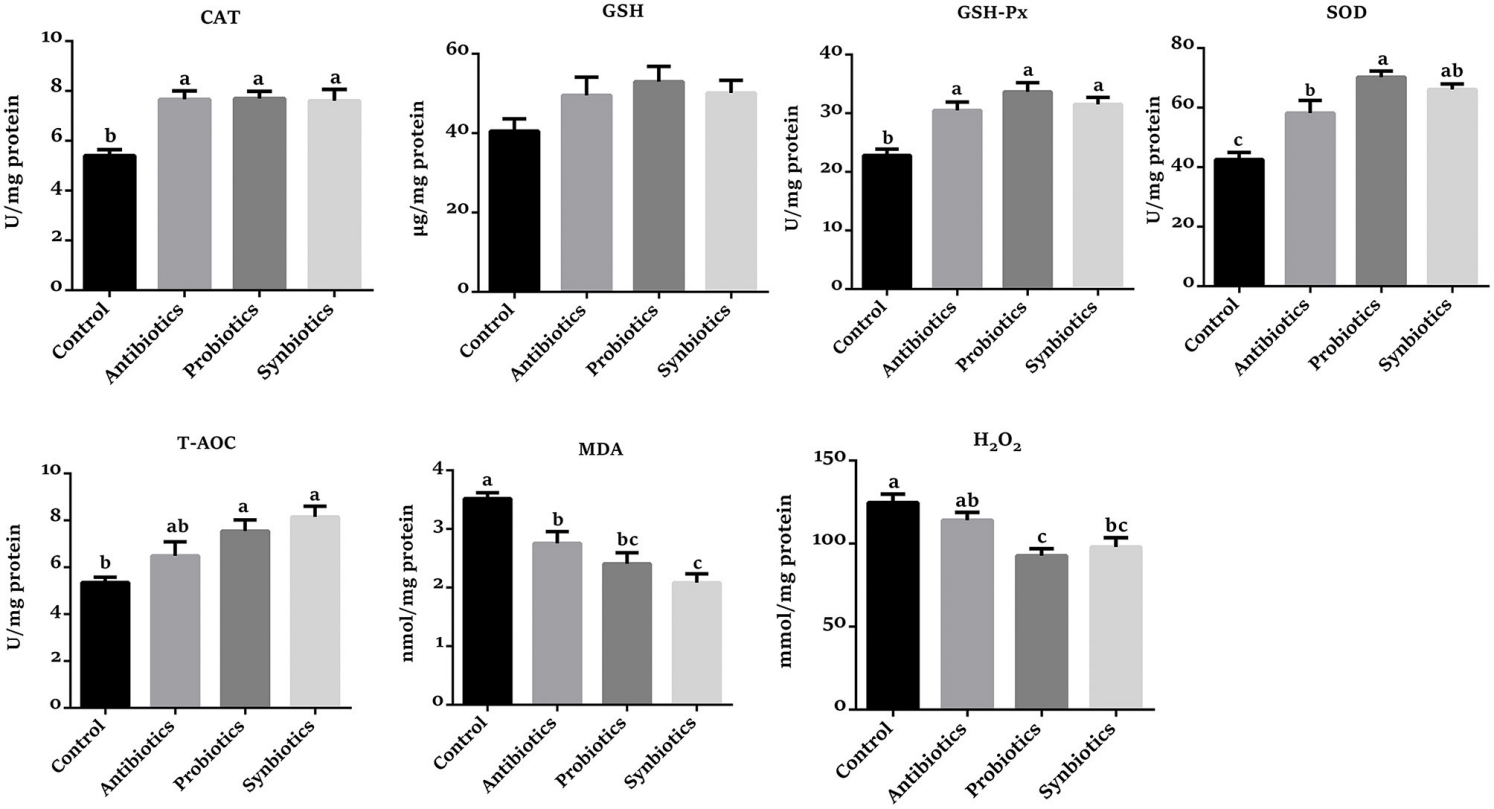
Effect of dietary probiotics or synbiotics supplementation during gestation, lactation, and nursery periods on the redox status in the colonic mucosa of weaned piglets. Data are means ± SEM (*n* = 8). a–c represents statistically significant differences among the four groups (*P* < 0.05). CAT, catalase; GSH, glutathione; GSH-Px, glutathione peroxidase; SOD, superoxide dismutase; T-AOC, total antioxidant capacity; MDA, malondialdehyde.

As presented in Figure 2, the CAT, GSH-Px, SOD, and T-AOC levels were higher (*P* < 0.05) in the probiotics group, while the concentrations of MDA and H_2_O_2_ were lower (*P* < 0.05) compared with the control group. The CAT, GSH-Px, SOD, and T-AOC levels were increased (*P* < 0.05), while the concentrations of H_2_O_2_ and MDA were decreased (*P* < 0.05) in the synbiotics group compared with the control group. Similarly, the SOD, CAT, and GSH-Px activities were increased (*P* < 0.05) while the concentrations of H_2_O_2_ and MDA were decreased (*P* < 0.05) in the antibiotics group. Compared with the antibiotics group, the SOD activity was increased (*P* < 0.05) while the H_2_O_2_ concentration was decreased (*P* < 0.05) in the probiotics group; the MDA concentration was lower (*P* < 0.05) in the synbiotics group.

### sIgA and Cytokine Concentrations in Colonic Mucosa

**Figure 3:**
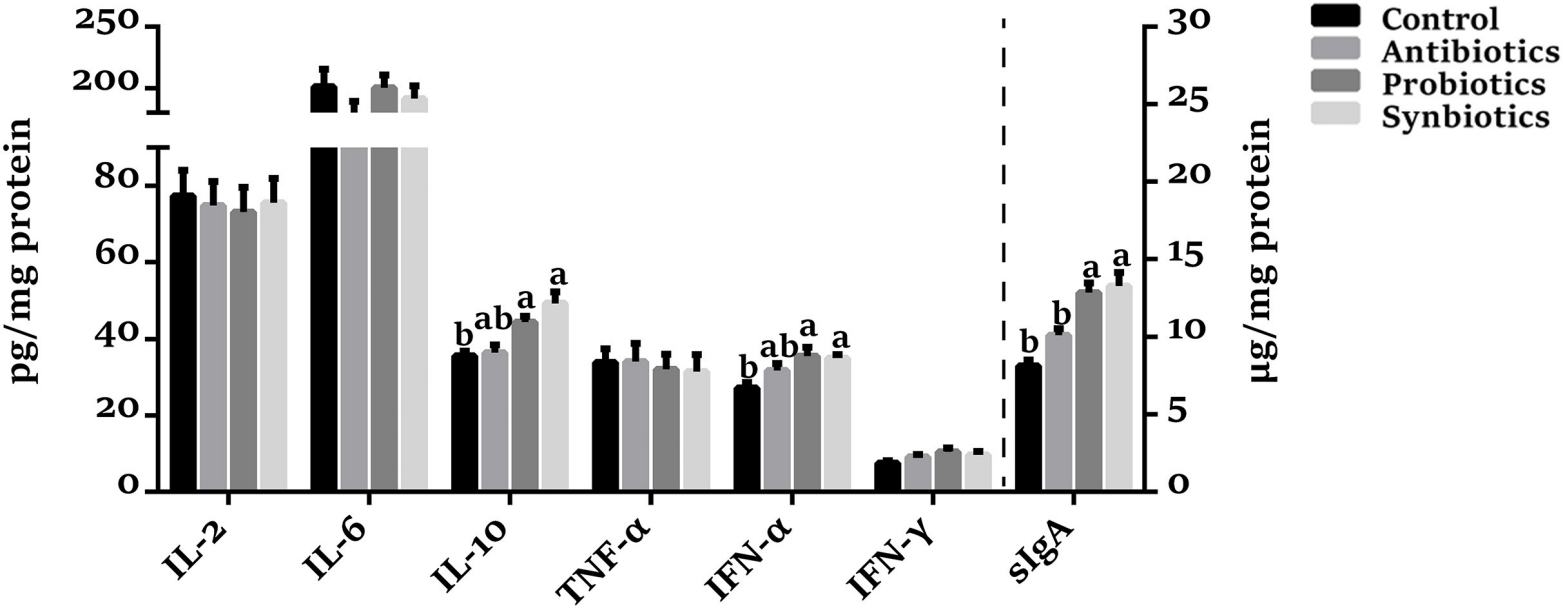
Effect of dietary probiotics or synbiotics supplementation during gestation, lactation, and nursery periods on the concentrations of cytokines and sIgA in the colonic mucosa of weaned piglets. Data are means ± SEM (*n* = 8). a,b represents statistically significant differences among the four groups (*P* < 0.05). IL-2, Interleukin-2; IL-6, Interleukin-6; IL-10, Interleukin-10; TNF-α, Tumor necrosis factor-alpha; IFN-α, Interferon-alpha; IFN-γ, Interferon-gamma; sIgA, secretory immunoglobulin A.

As shown in Figure 3, no significant differences in IL-2, IL-6, and TNF-α concentrations were observed among the four groups. Compared with the control group, the colonic sIgA, IL-10, and IFN-α concentrations were higher (*P* < 0.05), and the IFN-γ concentration tended to increase (*P* = 0.074) in the probiotics group. Synbiotics supplementation increased (*P* < 0.05) the sIgA, IL-10, and IFN-α concentrations compared with the control group. Dietary antibiotics supplementation tended to increase (*P* = 0.099) sIgA concentration, but not cytokine concentrations. Compared with the antibiotics group, the concentrations of sIgA (*P* < 0.05) and IL-10 (*P* = 0.067) were increased in the probiotics and synbiotics groups.

### Redox Status in Plasma

**Figure 4:**
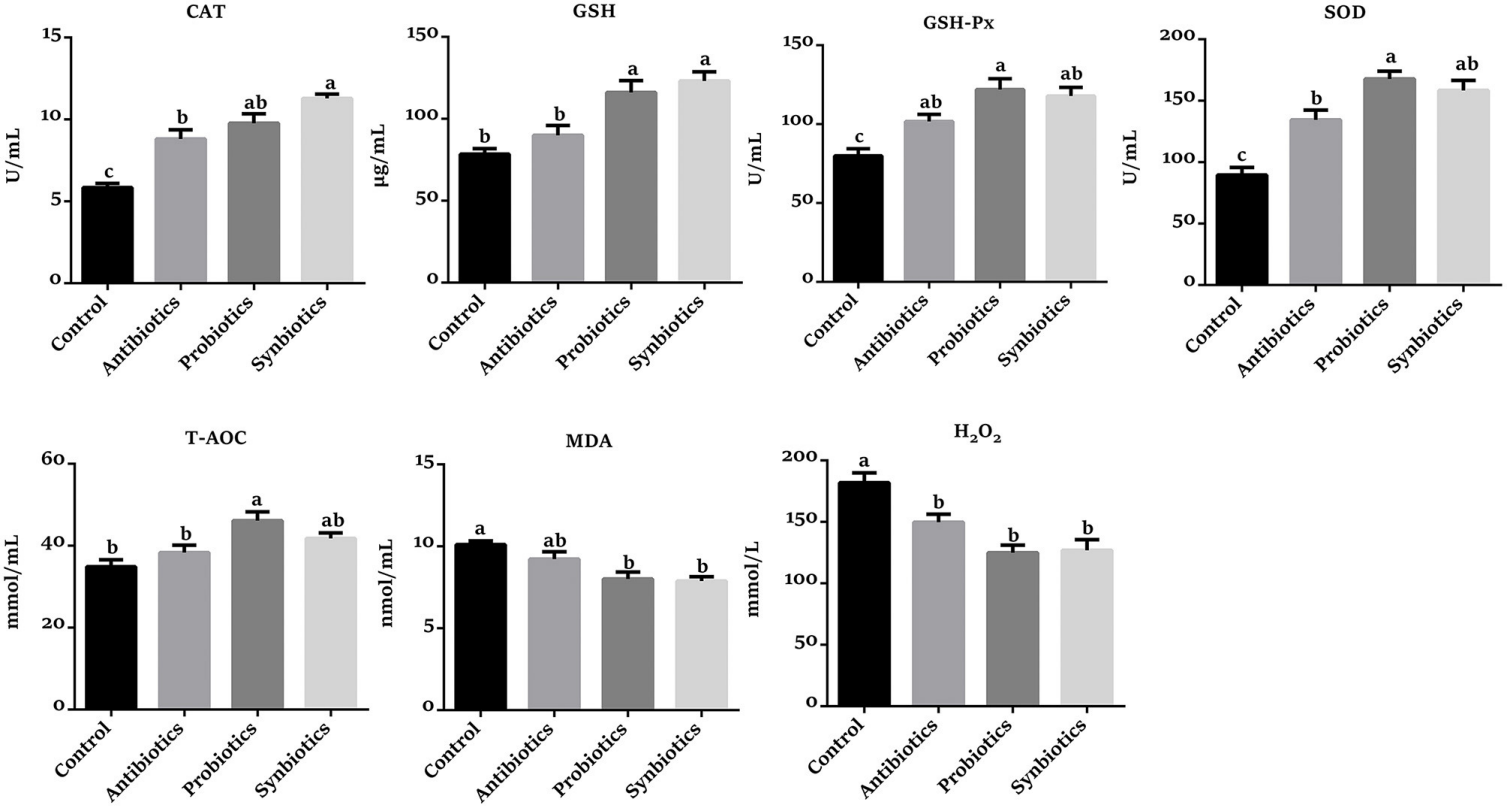
Effect of dietary probiotics or synbiotics supplementation during gestation, lactation, and nursery periods on the redox status in the plasma of weaned piglets. Data are means ± SEM (*n* = 8). a–c represents statistically significant differences among the four groups (*P* < 0.05). CAT, catalase; GSH, glutathione; GSH-Px, glutathione peroxidase; SOD, superoxide dismutase; T-AOC, total antioxidant capacity; MDA, malondialdehyde.

As presented in Figure 4, compared with the control group, the CAT, GSH, GSH-Px, SOD, and T-AOC levels were increased (*P* < 0.05), while the MDA and H_2_O_2_ concentrations were decreased (*P* < 0.05) in the probiotics group. Similarly, the CAT, GSH, GSH-Px, and SOD levels were significantly higher (*P* < 0.05), while the MDA and H_2_O_2_ concentrations were remarkably lower (*P* < 0.05) in the synbiotics group compared with the control group. Likewise, the CAT, GSH-Px, and SOD activities were increased (*P* < 0.05) while the H_2_O_2_ concentration was decreased (*P* < 0.05) in the antibiotics group compared with the control group. Compared to the antibiotics group, the GSH, SOD, and T-AOC levels were increased (*P* < 0.05) and GSH-Px tended to increase (*P* = 0.083) in the probiotics group, as well as the CAT and GSH activities in the synbiotics group.

### Immunoglobulin and Cytokine Concentrations in Plasma

**Figure 5:**
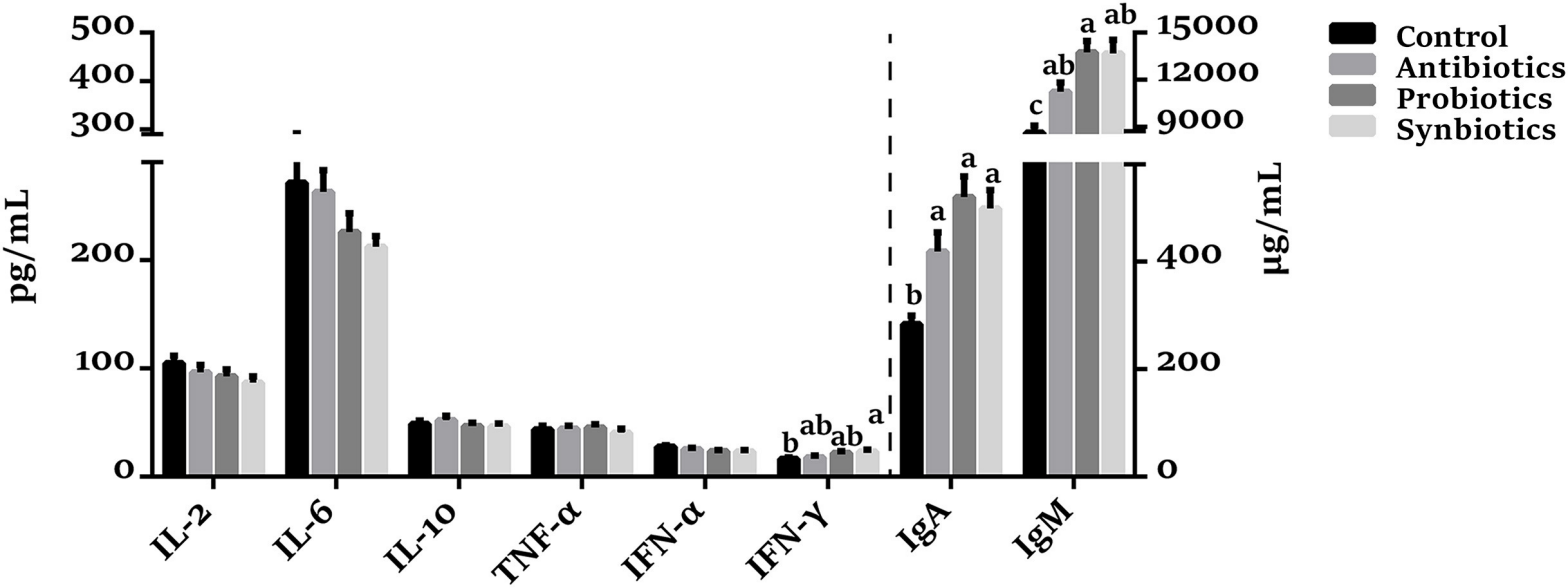
Effect of dietary probiotics or synbiotics supplementation during gestation, lactation, and nursery periods on the cytokines, IgA, and IgM concentrations in plasma of weaned piglets. Data are means ± SEM (*n* = 8). a–c represents statistically significant differences among the four groups (*P* < 0.05). IL-2, Interleukin-2; IL-6, Interleukin-6; IL-10, Interleukin-10; TNF-α, Tumor necrosis factor-alpha; IFN-α, Interferon-alpha; IFN-γ, Interferon-gamma; IgA, immunoglobulin A; IgM, immunoglobulin M.

As shown in Figure 5, there was no significant difference in IL-2, IL-6, IL-10, and TNF-α concentrations among the four groups. Compared with the control group, the concentrations of IgA (*P* < 0.05), IgM (*P* < 0.05), and INF-γ (*P* = 0.084) were increased in the probiotics group. Similarly, the IgA, IgM, and INF-γ concentrations were increased (*P* < 0.05) while the IFN-α concentration was decreased (*P* = 0.095) in the synbiotics group. The IgA and IgM concentrations were increased (*P* < 0.05) in the antibiotics group compared with the control group. Interestingly, the IgM concentration was higher (*P* = 0.060) in the probiotics group than in the antibiotics group.

### Correlation of Colonic Microbiota Abundance and Immune and Redox Indexes Levels in Colon and Plasma

**Figure 6:**
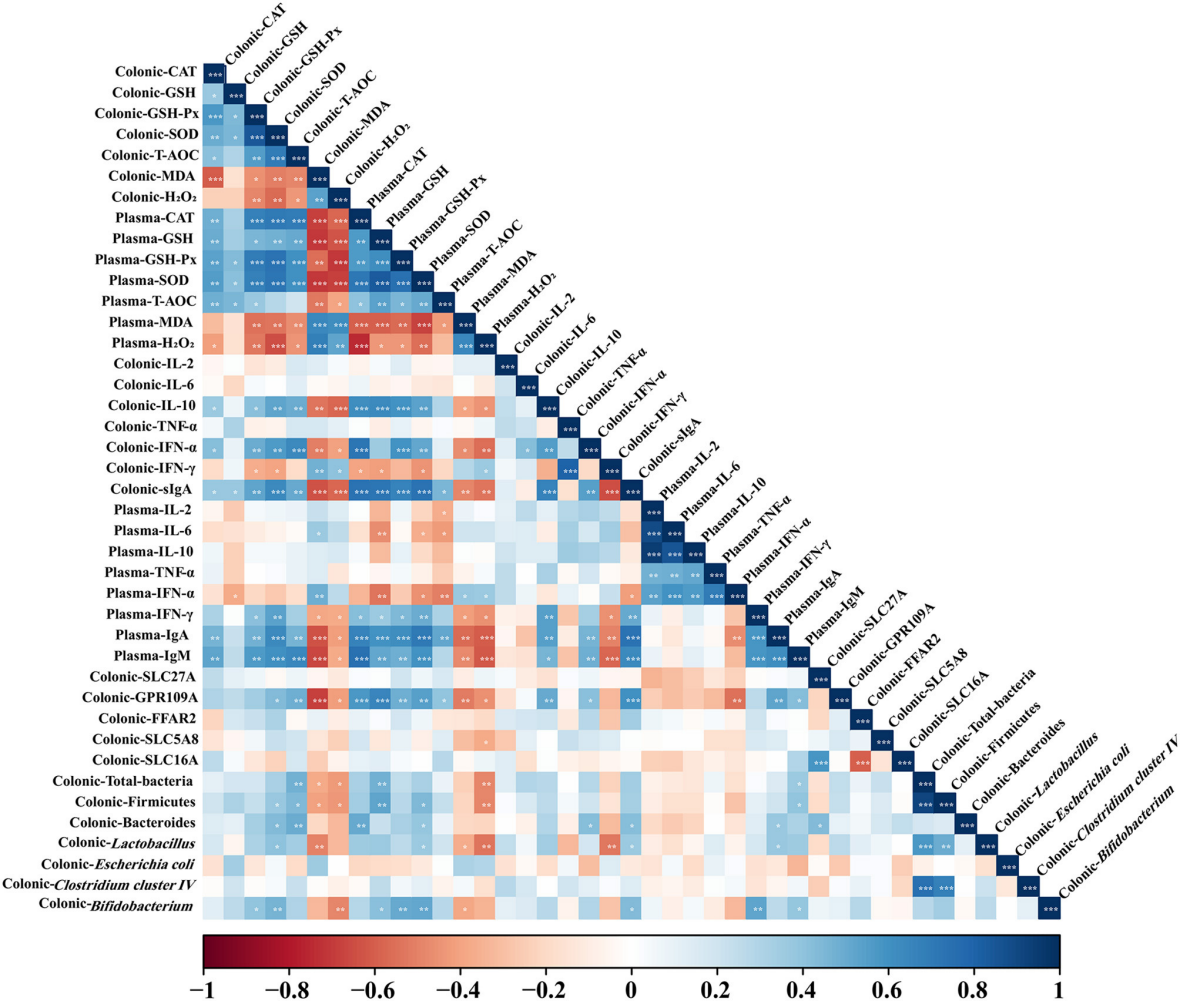
Correlation analysis of colonic dominant microbial abundance, immune marker, and antioxidant indexes levels in colon and plasma. The R package of “corroplot” was used for generating the heat maps. The blue represents a significant positive correlation, and the red represents a significant negative correlation. Asterisks indicate statistically significant difference: *P < 0.05; **P < 0.01; ***P < 0.001. CAT, Catalase; GSH, glutathione; GSH-Px, glutathione peroxidase; IFN-α, Interferon-alpha; IFN-γ, Interferon-gamma; IgA, immunoglobulin A; IgM, immunoglobulin M; IL-2, Interleukin-2; IL-6, Interleukin-6; IL-10, Interleukin-10; MDA, malondialdehyde; sIgA, secretory immunoglobulin A; SOD, superoxide dismutase; T-AOC, total antioxidant capacity; TNF-α, Tumor necrosis factor-alpha.

As shown in Figure 6, total bacteria abundance was positively correlated (*P* < 0.05) with colonic T-AOC level and plasma IgM and GSH levels, but negatively correlated (*P* < 0.05) with colonic H_2_O_2_ and MDA concentrations and plasma H_2_O_2_ concentration. *Firmicutes* abundance was positively correlated (*P* < 0.05) with colonic SOD and T-AOC levels and plasma GSH and SOD levels, but negatively correlated (*P* < 0.05) with colonic MDA and H_2_O_2_ concentrations and plasma H_2_O_2_ concentration. *Bacteroidetes* abundance was positively correlated (*P* < 0.05) with colonic sIgA, IFN-α, SOD, and T-AOC levels and plasma IgA, SOD, and CAT levels. *Lactobacillus* abundance was positively correlated (*P* < 0.05) with colonic sIgA and SOD levels and plasma IgA and SOD levels, but negatively correlated (*P* < 0.05) with colonic MDA concentration and plasma MDA and H_2_O_2_ concentrations. *Bifidobacterium* abundance was positively correlated (*P* < 0.05) with colonic sIgA, GSH-Px, and SOD levels and plasma IgM, IFN-γ, GSH, GSH-Px, and SOD levels, but negatively correlated (*P* < 0.05) with plasma MDA and colonic H_2_O_2_ concentrations. Colonic sIgA and IL-10 concentrations were positively correlated (*P* < 0.05) with plasma IgA, IgM, and IFN-γ concentrations. Colonic IFN-α concentration showed positive correlation (*P* < 0.05) with plasma IgA and IgM concentrations. Colonic CAT, GSH-Px, SOD, and T-AOC levels showed a positive correlation (*P* < 0.05) with plasma CAT, GSH, GSH-Px, and SOD levels, but were negatively correlated (*P* < 0.05) with plasma MDA and H_2_O_2_ concentrations. Colonic MDA and H_2_O_2_ concentrations were negatively correlated (*P* < 0.05) with plasma CAT, GSH, GSH-Px, SOD, and T-AOC levels, but positively correlated (*P* < 0.05) with plasma MDA and H_2_O_2_ concentrations. Colonic CAT, GSH-Px, SOD, and T-AOC levels showed a positive correlation (*P* < 0.05) with colonic IL-10, IFN-α, and sIgA concentrations and plasma IgA and IgM concentrations. Colonic MDA and H_2_O_2_ concentrations were negatively correlated (*P* < 0.05) with colonic IL-10, IFN-α, and sIgA concentrations and plasma IgA, IgM, and IFN-γ concentrations.

## Discussion

The dietary intervention of intestine microbiota composition during pregnancy and lactation periods have been proposed as a possible strategy to regulate the immune system and intestinal microbiota in offspring (6, 19–21). Probiotics intervention on sows has been reported to influence the development of intestinal microbiota of piglets (12, 22). In this context, the present study determined the effect of dietary probiotics or synbiotics supplementation during gestation, lactation, and nursery periods on colonic microbiota population, antioxidant capacity, and immune function in weaned piglets. The results showed that dietary probiotics or synbiotics supplementation during gestation, lactation, and nursery periods could increase the abundances of generally beneficial bacteria (*Bifidobacterium* and *Lactobacillus*), decrease *E. coli*, and enhance the immunity and antioxidant capacity in the colon and plasma of offspring weaned piglets.

Dietary probiotics or synbiotics supplementation is usually considered an effective way to positively modulate the intestinal microbiota in weaned piglets (9, 23). Based on the colonic microbiota analyses, we found that dietary probiotics or synbiotics supplementation significantly increase the relative abundances of *Firmicutes* and *Bacteroidetes*. The *Firmicutes* is the dominant microbiota in the gut that was reported to affect the metabolic capacity of the gut microbiota and enhances the body's ability to acquire energy from diets (24). *Bacteroidetes* generally produce butyrate, an important energy source for intestinal epithelial cells, and promote the proliferation of crypts (25). Our findings suggested that long-term dietary probiotics or synbiotics supplementation increased the bacteria associated with energy metabolism, which might benefit the energy recycling and intestinal development of weaned piglets. We also found that the abundances of *Bifidobacterium and Lactobacillus* were significantly increased in probiotics or synbiotics-treated piglets and numerically higher in antibiotics-treated groups. These two bacteria, as potential probiotics, were reported to be the health-promoting bacterium (26). An increase in *E. coli* amount was shown to be associated with the development of intestinal disease (27). The present study also showed that synbiotics treatment decreased the *E. coli* abundance, suggesting that the synbiotics addition can protect the gut from bacterial infection. Moreover, *Bacteroidetes* was reported to limit the intestinal colonization of potentially pathogenic bacteria (28), which was consistent with a decrease in *E. coli*.

The colon contains the trillions of commensal microbiota which is involved in the fermentation of indigestible carbohydrates and proteins (29). The fermentation products, such as short-chain fatty acid (SCFA), enter the colon cells via special receptors and transporters and present important roles in gut health status. The SCFA absorption can be greatly enhanced by two different solute transporters, the proton-coupled monocarboxylate-transporter 1 (MCT1/*SLC16A1*) and sodium-coupled monocarboxylate-transporter 1 (SMCT1/*SLC5A8*) (30, 31). Alternatively, GPR109A, FFAR3 (GPR41), and FFAR2 (GPR43) as the SCFA receptors are activated mainly by SCFA (32). In the present study, the GPR109A expression was upregulated in the probiotics-treatment group, suggesting that the colonic absorption function for SCFA was enhanced.

It is universally accepted that antioxidant enzymes, including CAT, GSH, GSH-Px, and SOD, are the important components in the body's antioxidant system, as they can catalyze a variety of chemical reactions related to reactive oxygen species and play a vital role in self-defense (33). MDA is a marker of lipid peroxidation, the concentration of which directly reflects the degree of oxidative injury (34). Probiotic species defense against the damage of ROS by involving both enzymatic (SOD and CAT) and non-enzymatic components. For example, *L. fermentum* treatment could increase T-AOC activity, decrease the ROS level by NOS1-mediating NO production in the intestinal epithelial cell (35); and *L. delbrueckii* supplementation elevated T-AOC and GSH levels while reduced MDA concentration in the intestine of LPS-changed piglets (36); long-term administration of *L. Plantarum* B90 and *S. cerevisiae* P11 increased the levels of CAT, T-AOC, SOD, GSH, and GSH-Px while decreased MDA concentration in the colon and plasma of piglets. Research has shown that oral supplements of combined fructo- and xylo-oligosaccharides during the perinatal period significantly diminished SOD and CAT activities in the brain of maternal and offspring in rats (37). In the present study, the synbiotics (XOS + compound probiotics) treatment also significantly improves the antioxidant capacity in the colon and plasma of weaned piglets. In addition, our study also showed that antibiotics treatment can improve colonic and plasma CAT and GSH-Px levels while decreased colonic MDA and plasma H_2_O_2_ concentrations which is consistent with the previous study (38). Both *Lactobacillus* and *Bifidobacterium*, as commonly used probiotics, play a vital role in improving the antioxidant capacity of the host. *Bifidobacterium animalis* 01 were reported to scavenge ROS, including hydroxyl radical and superoxide anion while enhancing the antioxidase activity of mice (39). Lactic acid bacteria (LAB) strains can scavenge free radicals, including peroxide radicals, superoxide anions, and hydroxyl radicals (40, 41). Correlation analysis revealed that the *Lactobacillus* abundance was positively correlated with the SOD activity in colon and plasma, while negatively correlated with colonic MDA concentration and plasma MDA and H_2_O_2_ concentrations, suggesting that the enhanced antioxidant ability in antibiotics, probiotics, and synbiotics groups may be due to the increased colonic *Lactobacillus* abundance. In addition, the plasma GSH, GSH-Px, and SOD levels and colonic GSH-Px and SOD activities were positively correlated while colonic H_2_O_2_ and plasma MDA concentrations were negatively correlated with *Bifidobacterium* abundance, suggesting that the enhanced antioxidant ability in the antibiotics, probiotics, and synbiotics groups may partly be due to the increased colonic *Bifidobacterium* abundance. Based on these findings, we hypothesized that dietary antibiotics, probiotics, and synbiotics supplementation enhance the antioxidant ability of the host partly resulting in alteration of intestine microbiota composition.

Several studies demonstrated the immune modulator properties of probiotic bacteria. *Bifidobacterium lactis* (*B. lactis*) is the most widely studied strain among *Bifidobacterium* species, which has been reported to enhance T lymphocytes and NK cell activity (42). Daily supplementing *B. lactis* HN019 to pregnant women increases cord blood and blood IFN-γ level compared with the placebo group (43). Correlation analysis revealed that plasma IFN-γ was positively correlated with colonic *Bifidobacterium* abundance, suggesting that an increase in plasma IFN-γ level in the probiotics and synbiotics groups may be due to the increase in colon *Bifidobacterium*. The increased colonic inflammatory cytokines may decrease susceptibility to infection by pathogens, which is consistent with the reduced *E. coli* abundance.

sIgA is the major regulator in the local immune barrier of the intestine, contributing to maintaining intestinal homeostasis (44). Therefore, intestinal sIgA level was used as an indicator to evaluate the immunity function of the intestinal mucosa. A previous study showed that probiotics could induce intestine mucus production and macrophage activation and elevated the sIgA and peripheral immunoglobulins levels (45). In the present study, probiotics and synbiotics treatment significantly increased the sIgA concentration, and dietary antibiotics supplementation tended to increase sIgA concentration in the colonic mucosa, implicating that these additives could enhance the intestinal immune response. Previous studies also reported that *Bifidobacterium* and *L. frumenti* could induce the production of intestinal sIgA (8, 46). Correlation analysis revealed that the sIgA concentration was positively correlated with *Bifidobacterium* and *Lactobacillus* abundances, suggesting that the increased colonic sIgA level in the antibiotics, probiotics, and synbiotics groups may be due to the increased benefit bacteria in the colon. IgA and IgG, as the major immunoglobulins in plasma, are the main components of humoral immunity, which play a vital role in protecting the host against invading pathogens (47). In the present study, dietary antibiotics, probiotics, or synbiotics supplementation also increased plasma IgA and IgM concentrations compared with the control group, which may result from *Bifidobacterium bifidum* (48) and *Bacillus subtilis* (49), due to of which can increase the level of serum immunoglobulin. The underlying mechanism maybe include the promotion of immunologic barrier and alteration in gut microbiota.

## Conclusion

The present study showed that dietary probiotics or synbiotics supplementation to sows and their offspring could increase the abundances of generally beneficial bacteria (*Bifidobacterium* and *Lactobacillus*), decrease potentially harmful bacteria (*E. coli*), enhance colonic absorption function for SCFAs, and improve the immune response and antioxidant capacity in offspring piglets. The changes in intestinal microbiota are correlated with alterations of immunoglobulins and cytokines concentrations and antioxidant capacity. These findings provided a potential approach to reduce the risk of intestinal dysfunction in offspring piglets.

## Data Availability Statement

The original contributions presented in the study are included in the article/supplementary materials, further inquiries can be directed to the corresponding author/s.

## Ethics Statement

The animal study was reviewed and approved by the Animal Care and Use Committee of the Institute of Subtropical Agriculture, Chinese Academy of Sciences.

## Author Contributions

XK and QH designed the experiment. KW, QZ, MS, LX, MA, and YZ carried out the animal experiment, sample collection, and sample analysis. KW performed the statistical analyses. KW, XK, and QH wrote the manuscript. All authors read and approved the final manuscript.

## Conflict of Interest

The authors declare that the research was conducted in the absence of any commercial or financial relationships that could be construed as a potential conflict of interest.
