# Supplementary material for: Dietary Probiotics or Synbiotics Supplementation During Gestation, Lactation, and Nursery Periods Modifies Colonic Microbiota, Antioxidant Capacity, and Immune Function in Weaned Piglets
Source: Front Vet Sci. 2020 Dec 14;7:597832. doi: 10.3389/fvets.2020.597832 (PMC7767837; doi:10.3389/fvets.2020.597832)
Supplement: Supplementary file 1 [file Table_1.pdf]

Supplementary Table 1 Primer sequences used for bacteria 16S rRNA

Target	Sequence 5’-3’	Product size (bp)	Reference
Total bacteria	F: GTGSTGCAYGGYYGTCGTCA	123	[1]
	R: ACGTCRTCCMCNCCTTCCTC		
Bacteroidetes	F: GGARCATGTGGTTTAATTCGATGAT	126	[2]
	R: AGCTGACGACAACCATGCAG		
Firmicutes	F: GGAGYATGTGGTTTAATTCGAAGCA	126	[3]
	R: AGCTGACGACAACCATGCAC		
Bifidobacterium	F: TCGCGTCYGGTGTGAAAG	128	[4]
	R: GGTGTTCTTCCCGATATCTACA		
Clostridium cluster IV	F: GCACAAGCAGTGGAGT	240	[5]
	R: CTTCCTCCGTTTTGTCAA		
Escherichia coli	F: CATGCCGCGTGTATGAAGAA	95	[6]
	R: CGGGTAACGTCAATGAGCAAA		
Lactobacillus	F: AGCAGTAGGGAATCTTCCA	345	[6]
	R: ATTCCACCGCTACACATG		

References

1. Maeda H, Fujimoto C, Haruki Y, Maeda T, Kokeguchi S, Petelin M, et al. Quantitative real-time PCR using TaqMan and SYBR green for Actinobacillus actinomycetemcomitans, Porphyromonas gingivalis, Prevotella intermedia, tetQ gene and total bacteria. *FEMS Immunol Med Mic.* (2003) 39:81-86. doi: 10.1016/S0928-8244(03)00224-4.

2. Guo X, Xia X, Tang R, Zhou J, Zhao H, Wang K. Development of a real-time PCR method for Firmicutes and Bacteroidetes in faeces and its application to quantify intestinal population of obese and lean pigs. *Lett Appl Microbiol. (*2008) 47:367-373. doi: 10.1111/j.1472-765X.2008.02408.x.

3. Matsuki T, Watanabe K, Fujimoto J, Takada T, Tanaka R. Use of 16S rRNA gene-targeted group specific primers for real-time PCR analysis of predominant bacteria in human feces. *Appl Environ Microbiol.* (2004) 70:7220-7228. doi: 10.1128/AEM.70.12.7220-7228.2004.

4. Huijsdens XW, Linskens RK, Mak M, Meuwissen SG, Vandenbroucke-Grauls CM, Savelkoul PH. Quantification of bacteria adherent to gastrointestinal mucosa by real-time PCR. *J Clin Microbiol.* (2002) 40:4423-4427. doi: 10.1128/jcm.40.12.4423-4427.2002.

5. Khafipour E, Li S, Plaizier JC, Krause DO. Rumen microbiome composition determined using two nutritional models of subacute ruminal acidosis. *Appl Environ Microbiol.* (2009) 75:7115-7124. doi: 10.1128/AEM.00739-09.

6. Walker AW, Ince J, Duncan SH, Webster LM, Holtrop G, Ze X, et al. Dominant and diet-responsive groups of bacteria within the human colonic microbiota. *ISME J*. (2011) 5:220-230. doi: 10.1038/ismej.2010.118.
