# Supplementary material for: Dietary Probiotics or Synbiotics Supplementation During Gestation, Lactation, and Nursery Periods Modifies Colonic Microbiota, Antioxidant Capacity, and Immune Function in Weaned Piglets
Source: Front Vet Sci. 2020 Dec 14;7:597832. doi: 10.3389/fvets.2020.597832 (PMC7767837; doi:10.3389/fvets.2020.597832)
Supplement: Supplementary file 2 [file Table_2.pdf]

Supplementary Table 2 Primer sequences for colonic mucosa short-chain fatty acid transporters and receptors mRNA analysis

Genes	Primers (5′-3′)	Size (bp)	Accession NO.
β-Actin	F: GATCTGGCACCACACCTTCTACAAC<br>R: TCATCTTCTCACGGTTGGCTTTGG	107	XM_021086047.1
FFAR2	F: CCTCATGGGTTTCGGCTTCTACAG<br>R: GCCACCAGAGCAGCAATCACTC	150	NM_001278758.1
SLC5A8	F: CTGCTCATCTCTGCGGTCATTGG<br>R: AGCTGGCAGTGAGGGACATCG	130	NM_001291414.1
SLC16A1	TTCATCGGCATGGGCATCAACTAC<br>TCAGCAGCGTCTATACTGGTCTCC	107	NM_001128445.1
SLC27A4	ATGGTGCTCTTGAAGGTGAAGGC<br>CTGGACGGTGGCAGCGAATAAG	87	XM_021069609.1
GPR109A	GAGGGCCATCAACTTCGTCA<br>AGTTGGGGAAAGATGGGCTG	230	XM_021072989.1
