# Supplementary material for: Dietary Probiotics or Synbiotics Supplementation During Gestation, Lactation, and Nursery Periods Modifies Colonic Microbiota, Antioxidant Capacity, and Immune Function in Weaned Piglets
Source: Front Vet Sci. 2020 Dec 14;7:597832. doi: 10.3389/fvets.2020.597832 (PMC7767837; doi:10.3389/fvets.2020.597832)
Supplement: Supplementary file 3 [file Image_1.pdf]

Supplementary Figure

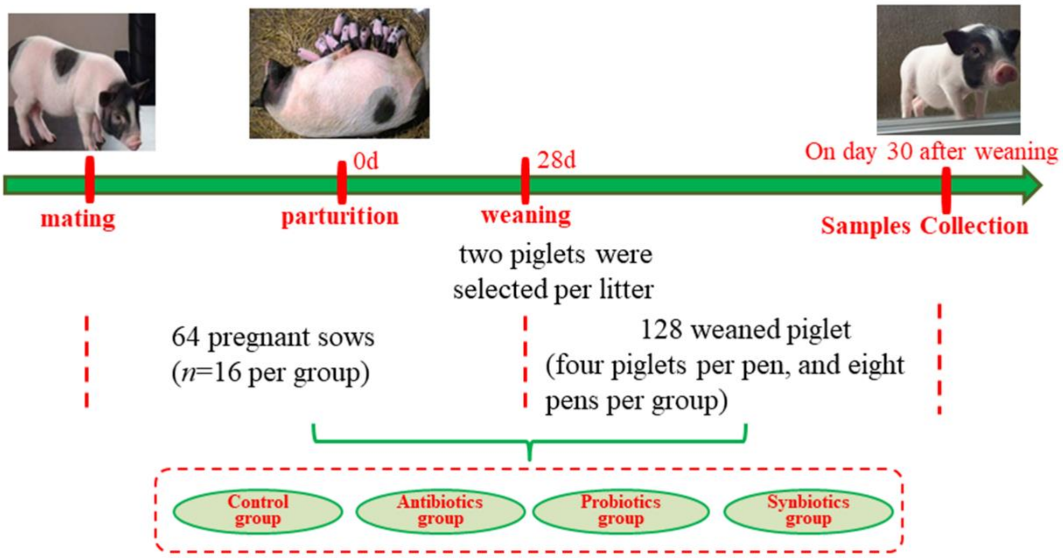

Supplementary Figure 1. Schematic showing the experimental design of this study.
